# Proteomic Profiling of the Amniotic Fluid to Detect Inflammation, Infection, and Neonatal Sepsis

**DOI:** 10.1371/journal.pmed.0040018

**Published:** 2007-01-16

**Authors:** Catalin S Buhimschi, Vineet Bhandari, Benjamin D Hamar, Mert-Ozan Bahtiyar, Guomao Zhao, Anna K Sfakianaki, Christian M Pettker, Lissa Magloire, Edmund Funai, Errol R Norwitz, Michael Paidas, Joshua A Copel, Carl P Weiner, Charles J Lockwood, Irina A Buhimschi

**Affiliations:** 1 Department of Obstetrics, Gynecology and Reproductive Science, Yale University School of Medicine, New Haven, Connecticut, United States of America; 2 Department of Pediatrics, Division of Perinatal Medicine, Yale University School of Medicine, New Haven, Connecticut, United States of America; 3 Department of Obstetrics and Gynecology, University of Kansas School of Medicine, Kansas City, Kansas, United States of America; Imperial College London, United Kingdom

## Abstract

**Background:**

Proteomic analysis of amniotic fluid shows the presence of biomarkers characteristic of intrauterine inflammation. We sought to validate prospectively the clinical utility of one such proteomic profile, the Mass Restricted (MR) score.

**Methods and Findings:**

We enrolled 169 consecutive women with singleton pregnancies admitted with preterm labor or preterm premature rupture of membranes. All women had a clinically indicated amniocentesis to rule out intra-amniotic infection. A proteomic fingerprint (MR score) was generated from fresh samples of amniotic fluid using surface-enhanced laser desorption ionization (SELDI) mass spectrometry. Presence or absence of the biomarkers of the MR score was interpreted in relationship to the amniocentesis-to-delivery interval, placental inflammation, and early-onset neonatal sepsis for all neonates admitted to the Newborn Special Care Unit (*n* = 104). Women with “severe” amniotic fluid inflammation (MR score of 3 or 4) had shorter amniocentesis-to-delivery intervals than women with “no” (MR score of 0) inflammation or even “minimal” (MR score of 1 or 2) inflammation (median [range] MR 3–4: 0.4 d [0.0–49.6 d] versus MR 1–2: 3.8 d [0.0–151.2 d] versus MR 0: 17.0 d [0.1–94.3 d], *p* < 0.001). Nonetheless, a “minimal” degree of inflammation was also associated with preterm birth regardless of membrane status. There was a significant association between the MR score and severity of histological chorioamnionitis (*r* = 0.599, *p* < 0.001). Furthermore, neonatal hematological indices and early-onset sepsis significantly correlated with the MR score even after adjusting for gestational age at birth (OR for MR 3–4: 3.3 [95% CI, 1.1 to 9.2], *p* = 0.03). When compared with other laboratory tests routinely used to diagnose amniotic fluid inflammation and infection, the MR score had the highest accuracy to detect inflammation (white blood cell count > 100 cells/mm^3^), whereas the combination of Gram stain and MR score was best for rapid prediction of intra-amniotic infection (positive amniotic fluid culture).

**Conclusions:**

High MR scores are associated with preterm delivery, histological chorioamnionitis, and early-onset neonatal sepsis. In this study, proteomic analysis of amniotic fluid was shown to be the most accurate test for diagnosis of intra-amniotic inflammation, whereas addition of the MR score to the Gram stain provides the best combination of tests to rapidly predict infection.

## Abstract 2

Proteomic analysis of amniotic fluid in addition to a Gram stain provides the best combination of tests to rapidly predict intrauterine infection.

## Editors' Summary

**Background.:**

A preterm delivery, or premature birth, is normally defined as one that occurs before 37 weeks after the last menstrual cycle (an average pregnancy lasts around 40 weeks). Premature birth is fairly common, with around 12% of births in the US fitting this definition. However, it has serious consequences, being responsible for around 70% of infant deaths and other adverse outcomes for the baby. It is not clear in all cases what directly causes premature birth or how to identify cases in which mother and child are at greater risk of serious outcomes. Evidence from case-control and other studies strongly suggests that infections of the uterus, placenta, or genital tract are associated with, and are likely to directly cause, premature deliveries. Such infections, even if they are “subclinical” (that is, they do not directly cause signs or symptoms that the doctor or patient would notice) cause inflammation in the affected tissues. Hence, it's possible that particular proteins or other molecules could provide a “signature” that would allow the inflammation to be picked up at an early stage.

**Why Was This Study Done?:**

If inflammation could be picked up early, this might help identify mothers at risk of having a preterm delivery, and even to pinpoint cases of very severe inflammation where the baby is more at risk of poor outcomes. The researchers involved in this study had already done previous work looking at protein profiles in the amniotic fluid (the liquid directly surrounding the developing fetus). They identified a set of four protein “markers” that were closely associated with inflammation in the amniotic fluid, and developed a score based on those proteins, which they termed the “Mass Restricted” (MR) score. The researchers showed that this score could accurately identify women at risk of preterm delivery. However, before using the protein marker score in clinical practice it is very important to really be sure it is a reliable diagnostic test for preterm birth and adverse outcomes resulting from preterm birth. Therefore the researchers wanted to find out whether MR scores were associated with the outcome of pregnancy; the presence of infection in the placenta, as detected through microscopic analysis of tissue; and sepsis (severe infection) in the newborn baby.

**What Did the Researchers Do and Find?:**

The study was based on findings from pregnant women presenting at the Yale-New Haven Hospital with symptoms of premature labor, who were all followed up to the point of delivery of the baby. In all cases the decisions about how to manage the pregnancy (for example, whether to deliver the baby or attempt to delay birth) were made by the woman and her physician, not by any procedures laid out in the research study. A total of 169 women were recruited into the study and had a sample of amniotic fluid taken as part of their routine clinical management. The researchers then analyzed this fluid to calculate the protein MR score, to look for evidence of bacterial infection, and also carried out standard laboratory tests. After childbirth the placenta was examined under the microscope to look for any evidence of inflammation. Finally, all babies were checked for any evidence of sepsis. The researchers found that, in line with findings from their previous studies, women with a higher MR score gave birth sooner. There also seemed to be a close agreement between the MR score and evidence of inflammation in the placenta, once it was analyzed under the microscope after birth. Furthermore, mothers with a high MR score were more likely to give birth to babies with suspected or confirmed sepsis. The researchers then compared the usefulness of the MR score against other potential tests for inflammation. Of all the tests compared, the MR score seemed to be the most accurate in predicting inflammation.

**What Do These Findings Mean?:**

This study showed that the MR score was** closely associated with a number of different indicators of poor outcome in preterm birth. These outcomes included sooner deliveries, sepsis in the baby, and inflammation in the placenta. In future, the MR score may provide a useful test for recognizing women at risk of preterm delivery and babies at risk of poor outcome. However, further evaluation of the test will still need to be done before it could become a standard procedure in the clinic.

**Additional Information.:**

Please access these Web sites via the online version of this summary at http://dx.doi.org/10.1371/journal.pmed.0040018.

- Information from the US National Institutes of Health on premature babies
- The March of Dimes is a US charity that funds research into prematurity
- Information from Wikipedia about proteomics the area of research used to develop the protein score examined here (note: Wikipedia is an online encyclopedia that anyone can edit)

## Introduction

Preterm delivery remains a major public health problem with lasting family and societal repercussions [1]. Prevention strategies have failed, and the prevalence of preterm birth in the United States rose to an unprecedented 12.3% in 2003 [2]. Preterm birth accounts for almost 70% of neonatal deaths and up to 75% of neonatal morbidity [1]. The consequences for the neonate can prove devastating when intra-amniotic infection is superimposed upon prematurity [1].

Both clinically symptomatic and asymptomatic intrauterine infection induce an intra-amniotic inflammatory response that includes the release of multiple cytokines and chemokines that in turn may trigger preterm contractions and/or rupture of the membranes [3]. Whereas an adequate innate and adaptive immune response is critical for survival of mother and child [4], a robust intrauterine inflammation remains a major risk for poor cognitive and neurodevelopmental outcome [5–7]. Thus, rapid and accurate recognition of inflammation in utero is critical if targeted interventions to change outcome are to be developed [8]. Unfortunately, this pursuit is complicated by the absence of rapid, sensitive, and specific diagnostic tests for intrauterine inflammation.

The science of proteomics has been applied to the search for biomarkers and generation of protein profiles that can rapidly aid the prediction, early diagnosis, and treatment of human diseases [9–13]. We have shown previously that proteomic mapping of amniotic fluid (AF) reveals a profile characteristic of intra-amniotic inflammation [14]. The sensitivity and specificity reached 100% in blinded testing [14]. Yet, the original enthusiasm for the “omics” discoveries was dampened by concern that these approaches are not truly disease specific and reproducible [15].

In this study, we sought to determine in a prospective and blinded fashion whether a previously identified proteomic profile comprised of four protein biomarkers (neutrophil defensins-1 and −2 and calgranulins A and C) is reproducible and maintains its highly accurate “signature” when compared to previously established or proposed markers of intra-amniotic fluid inflammation or infection: glucose, white blood cell (WBC) count, lactate dehydrogenase (LDH), Gram stain, interleukin-6 (IL-6), and matrix metalloprotease-8 (MMP-8). We further tested whether proteomic analysis of AF relates to pregnancy outcome, pathologic evaluation of the placenta, and early-onset neonatal sepsis.

## Methods

### Study Population and Specimens

AF was retrieved by ultrasound-guided amniocentesis from 169 consecutive women with singleton pregnancy following admission at Yale-New Haven Hospital with symptoms of premature labor (PTL), advanced cervical dilatation, and/or preterm premature rupture of membranes (PPROM). After the procedure, each woman was followed prospectively up to the point of delivery. The decision to recommend amniocentesis or delivery of the fetus was made by the primary clinical provider, independent of the research protocol. The Yale University Human Investigation Research Board approved the study (February 2004–April 2006). Written informed consent was obtained from all participants. Gestational age (GA) was established based on an ultrasonographic examination prior to 20 wk in all instances. Preterm labor was defined as regular uterine contractions associated with advanced cervical dilatation or effacement less than 37-wk gestation [16].

Membrane rupture was confirmed either by direct visualization of AF “pooling” on speculum examination, positive “nitrazine” and “ferning” tests, or by the infusion of indigo carmine into the amniotic sac. In patients with PPROM, cervical dilatation was interpreted based on visual inspection of the cervix at the time of the sterile speculum exam. Advanced cervical dilatation was defined as cervical dilatation of 3 cm or more. Clinical chorioamnionitis was diagnosed in the presence of maternal fever (>37.8 °C), uterine tenderness, foul-smelling AF or visualization of pus at the time of the speculum exam, maternal tachycardia (≥100 beats/min), or fetal tachycardia (≥160 beats/min) [16]. Induction of labor or a surgical delivery was performed for such clinical indications as a prolapsed umbilical cord or a GA of 34 wk or more [17], or AF laboratory results (glucose, LDH, WBC count, Gram stain, and microbial cultures) traditionally considered to indicate intra-amniotic inflammation/infection [16]. The decision for delivery was made by the primary physician who was blinded to all research test results.

In our prior descriptive study of the AF proteomic profile [14], intra-amniotic inflammation was defined as an AF WBC count greater than 100 cells/mm^3^. To validate and test the diagnostic performance of the proteomic profile in a prospective fashion, a similar definition of inflammation was maintained.

AF was cultured for aerobic and anaerobic bacteria, and *Ureaplasma* and *Mycoplasma* species. The clinical laboratory performed the glucose and LDH measurements, the WBC and red blood cell (RBC) counts, and the Gram staining. In four women, the WBC count could not be reported due to marked cellular destruction secondary to gross bacterial invasion of the AF. The clinical laboratory results were available to the primary care providers for clinical management.

A proteomic fingerprint, the Mass Restricted (MR) score, was immediately generated from the fresh AF using a single surface-enhanced laser desorption ionization time-of-flight (SELDI-TOF) mass spectrometer instrument [14]. The MR score provides qualitative information regarding the presence or absence of intra-amniotic inflammation [14]. The MR score ranges from 0 to 4, depending upon the presence or absence of each of the four protein biomarkers [14]. A categorical value of 1 is assigned if a particular peak is present and 0 if absent. A score of 3–4 indicates the presence of inflammation, whereas a score of 0–2 excludes it [14]. However, in the current investigation, we also stratified the study population based on the “severity” of inflammation (MR = 0 indicates “no” inflammation; MR = 1–2 indicates “minimal” inflammation; and MR = 3–4 indicates “severe” inflammation). One investigator (author IAB) performed all the protein chip array readings and scored all the samples “blindly,” being unaware of either clinical presentation or outcome. The MR score results were not used for clinical management. The remaining AF was centrifuged at 3,000 *g,* 4 °C for 10 min., aliquoted for research purposes, and stored at −80 °C until IL-6 and MMP-8 levels were measured using specific enzyme-linked immunosorbent assays (ELISAs) [18,19].

### Procedures

The methodology for generation of the MR score was previously described in detail [14,20]. The time necessary to prepare and report the MR score was documented electronically for each AF sample. We also investigated the minimum incubation time required to obtain an adequate SELDI spectrum for a reliable diagnosis of inflammation. For this purpose, eight AF samples from women with known MR scores of 4 and positive AF microbial cultures were placed on H4 ProteinChip arrays and incubated for 1 h as per general protocol or for shorter times (15, 30, or 45 min) before calculation of the MR scores.

AF glucose levels and total LDH activity were measured using an autoanalyzer (Roche/Hitachi SWA ISE 1800, http://www.roche-diagnostics.com). The lower limit of detection for this instrument is 2 mg/dl (0.11 mmol/l) for glucose and 1 U/l for the LDH. The coefficient of variation is less than 1% for glucose and less than 5% for the LDH. Because several AF glucose cutoffs (≤15 mg/dl or ≤10 mg/dl) have been reported in the literature to best predict intra-amniotic inflammation and/or infection, we analyzed the performance of both [21]. LDH levels of 419 U/l or greater were considered clinically suggestive of intra-amniotic inflammation and/or infection [22].

ELISAs for human IL-6 (Pierce-Endogen, http://www.piercenet.com) and MMP-8 (R & D Systems, http://www.rndsystems.com) were performed in duplicate according to the manufacturers' instructions by investigators unaware of the sample origin. The minimum detectable concentration for IL-6 was 1 pg/ml and less than 0.02 ng/ml for MMP-8. The inter- and intra-assay coefficients of variation were less than 10% for IL-6 and less than 6% for MMP-8, respectively. An AF concentration above 11.4 ng/ml for IL-6 and 23 ng/ml for MMP-8 were considered indicative of intra-amniotic inflammation or infection [18,19].

### Histological Evaluation of the Placenta for Acute Inflammation

At Yale New Haven Hospital, placental pathologic study is a routine part of the evaluation of a pregnancy complicated by PTL or PPROM. Because several patients enrolled in our study delivered at term or outside our hospital following discharge, tissue sections were available from only 117 patients who also provided AF samples. Sections were read by a perinatal pathologist unaware of the results of the proteomic profiling. From each placenta, sections of chorionic plate, extraplacental membranes, and umbilical cord were examined systematically for inflammation. Three histological stages of chorioamnionitis [23] (stage I: intervillositis, stage II: chorionic inflammation, and stage III: full thickness inflammation of both chorion and amnion) were complemented by the histological grading system devised by Salafia et al. which includes four grades of inflammation of the amnion, chorion-decidua, and umbilical cord [24].

### Evaluation of Early-Onset Neonatal Sepsis

Neonatal hematological indices and sepsis categorization were assessed as previously described [25,26] from blood specimens and cultures obtained immediately following delivery for all neonates admitted to the Newborn Special Care Unit (*n* = 104) by an investigator (author VB) unaware of the results of the proteomic profiling. All neonates underwent clinical and laboratory evaluations of sepsis. Clinical indications for early-onset neonatal sepsis included lethargy, apnea, respiratory distress, hypoperfusion, and shock. Early-onset neonatal sepsis was defined as the presence of confirmed or suspected sepsis at less than 3 d after birth. Confirmed sepsis was established when either the blood and/or cerebrospinal fluid culture was positive. Suspected sepsis was diagnosed in the presence of clinical suspicion of sepsis with support from other laboratory results. Early-onset neonatal sepsis was dichotomized into present (when sepsis was either confirmed or suspected) or absent, and coefficients of associations with other binary variables were calculated. Laboratory criteria were based on modification of the criteria of Rodwell et al. [25] when two or more of the following were observed: absolute neutrophil count (ANC) less than 7,500/ml or greater than 14,500/ml, absolute band count (ABC) greater than 1,500/ml, immature/total neutrophil ratio (I:T ratio) geater than 0.16, platelet count less than 150,000 cells/mm^3^, or an abnormal spinal tap [26]. All neonates with suspected or confirmed sepsis received antibiotic therapy.

### Statistical Analysis

Statistical analyses were performed with Sigma Stat, version 2.03 (SPSS, http://www.spss.com) and MedCalc (MedCalc Software, http://www.medcalc.be) statistical software. To avoid introducing spectrum bias [27], no patient or AF sample was excluded from the final analysis; a search for intra-amniotic inflammation/infection was made in each sample and a clinical decision taken independently following review of the laboratory results for each amniocentesis procedure. Test accuracy (cases correctly classified divided by total number of cases), sensitivity, specificity, positive and negative predictive values (PPV and NPV, respectively) were measured for each assay by examining the distribution of individual AF tests on receiver operator characteristic (ROC) plots. Because amniocenteses were performed across a wide range of gestation periods, the times from the amniocentesis to birth for the 169 patients were normalized to reflect pregnancy prolongation after amniocentesis as previously described [28]. The percent prolongation of pregnancy was calculated by dividing the amniocentesis-to-delivery interval by the number of days gestation at amniocentesis times 100. Kaplan-Meier probability plots were generated based on the duration from amniocentesis to delivery and their differences tested with the log-rank test (GraphPad Software, http://www.graphpad.com). Continuous data were compared with the Student *t*-test and one-way analysis of variance (ANOVA) followed by Student-Newman-Keuls tests (parametric) or Kruskal-Wallis on ranks followed by the Dunn tests (nonparametric) to adjust for multiple comparisons as appropriate. Comparisons between proportions were done with the Chi-square test or Chi-square test for trend (MedCalc). Two-by-two contingency tables were constructed and Chi-square analysis of independence was used to identify significant differences among test performances. The phi-coefficient of correlation (Φ), an index of association for binary data, was also calculated [29]. Variables (glucose, LDH, gram stain, IL-6, and MMP-8) were dichotomized based on the defined clinical thresholds. Multiple stepwise linear and logistic regression analyses were used to adjust *p*-values and odds ratios (ORs), respectively, for potential influences of gestational age or other parameters. A *p*-value of less than 0.05 was used to indicate significance.

## Results

### Characteristics of Women and at the Time of Sampling

The clinical characteristics of the women at amniocentesis are presented in Table 1. The median time from rupture to amniocentesis in the PPROM group was 9 h (range: 0.5–745.5 h). However, the amniocentesis-to-delivery interval was shorter for the PPROM in comparison to the intact group.

**Table 1:**
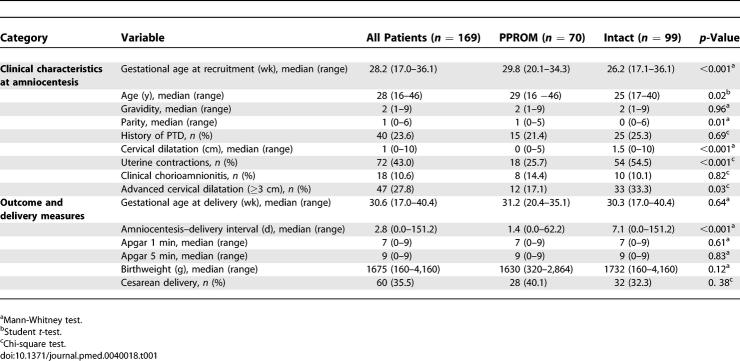
Characteristics of Women (*n* = 169) Who Had a Clinically Indicated Amniocentesis to Rule Out Inflammation and Infection, and Characteristics of Their Infants at Birth

### Biochemical and Proteomic Assessment of Intra-amniotic Inflammation and Infection

Women with PPROM had lower AF glucose levels compared to the intact group (Table 2). A higher percentage of women with PPROM had increased WBC and RBC counts and a higher prevalence of a positive microbial culture result. The most common isolates were Ureaplasma urealyticum (17/44), *Streptococcus* species (9/44), and *Bacteroides* species (5/44). Eleven women with positive AF cultures (25%) grew multiple microbes. Women with PPROM had elevated levels of MMP-8. More women with PPROM had a positive MR score (three or four biomarkers present).

**Table 2:**
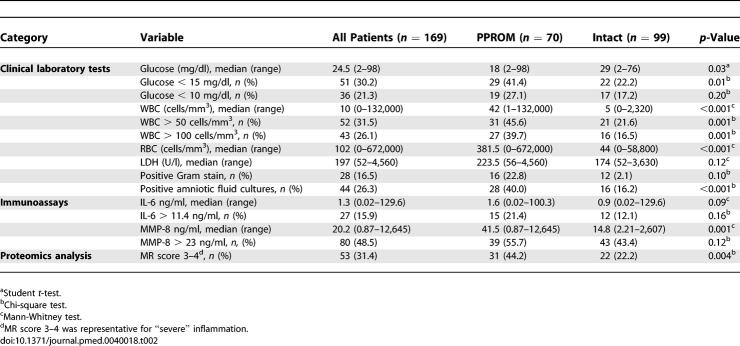
Results of the AF Clinical Laboratory Tests, ELISA for the IL-6 and MMP-8 Levels, and Proteomics Analysis in Our Study Population

### Biomarker Combinations and MR Scores

**Figure 1:**
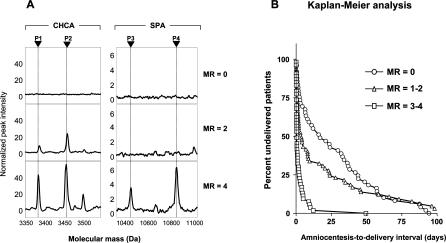
Proteomic Profiling of AF and Risk for Preterm Delivery (A) Representative SELDI-TOF mass spectrometry profiles of the AF based on the “severity” of inflammation (MR = 0 indicates “no” inflammation; MR = 2 indicates “minimal” inflammation; and MR = 3–4 indicates “severe” inflammation). Five microliters of diluted AF (1:10) was placed in duplicate on spots of H4 ProteinChip arrays (Ciphergen Biosystems, Fremont, California, United States) and incubated for 1 h. After 1 h, the AF samples were aspirated, and the spots were individually washed three times with 25% acetonitrile solution, air dried, and then overlaid with matrix solution (1 μl of 20% saturated solution of α-cyano-4 hydroxycinnamic acid [CHCA] on one array and two overlays of 1 μl of 50% saturated solution of sinnapinic acid [SPA] on the other array). The chips were air dried and then read in a Protein Biology System IIC (PBS IIC) SELDI-TOF mass spectrometer (Ciphergen Biosystems) using the ProteinChip software v3.1.1. The CHCA chip allows for the identification of defensins 2 (P1: 3,377.01 Da) and 1 (P2: 3,448.09 Da). The SPA chip allows for identification of the peaks corresponding to calgranulin C (P3: 10,443.85 Da) and calgranulin A (P4: 10,834.51 Da). The *x*-axis of the tracings represents the molecular mass in Daltons; the *y*-axis represents the relative peak intensity. (B) Cumulative probability of pregnancy maintenance for the 169 patients illustrating the duration from amniocentesis to delivery in women with MR scores of 0 (zero), MR scores of 1–2, and MR scores 3–4.

The distribution of MR scores in the 169 AF samples was: MR 0: *n* = 50 (29.6%); MR 1: *n* = 22 (13.0%); MR 2: *n* = 44 (26.0%); MR 3: *n* = 23 (13.6%); and MR 4: *n* = 30 (17.8%). All but two samples with an MR score of 1 reflected the presence of defensin 2 (P1, Figure 1A). Both exceptions had a peak corresponding to calgranulin A (P4). In most instances (41/44, 93.2%), an MR of 2 reflected the presence of defensin 1 (P2) and defensin 2 (P1). In only three instances, defensin 2 was accompanied by calgranulin A. In all but one case, an MR of 3 reflected the combination of defensins 1 and 2 and calgranulin C (P3). This suggests a sequential appearance of the biomarkers in women with intra-amniotic inflammation/infection.

### AF MR Scores, Severity of Inflammation, and Pregnancy Outcome

We used Kaplan-Meier analysis to examine the relationship between the severity of inflammation (MR score) and the duration of the amniocentesis-to-delivery interval. Women with “severe” inflammation (MR scores of 3 or 4) had shorter amniocentesis-to-delivery intervals than women with “no” (MR scores of 0) inflammation, or “minimal” (MR scores of 1 or 2) inflammation (median [range] MR 3–4: 0.4 d [0.0–49.6 d] versus MR 1–2: 3.8 d [0.0–151.2 d] versus MR 0: 17.0 d [0.1–94.3 d], *p* < 0.001) (Figure 1B). Nineteen women had MR scores of 3–4, but were all managed expectantly by the clinical provider who was unaware of the results of proteomic analysis. The median time from the amniocentesis to delivery in this subgroup of women was 0.7 d [0.0–49.6 d], which was not significantly different from that of the subgroup of women with MR 3–4 managed with indicated delivery (*p* = 0.73).

We further focused our analysis by stratifying it by membrane status (PPROM versus intact membranes). There was a significant relationship between the MR score and the amniocentesis-to-delivery interval in pregnancies complicated by PPROM. Women with PPROM and “severe” inflammation had significantly shorter amniocentesis-to-delivery intervals compared with women with both “minimal” inflammation and “no” inflammation (MR 3–4: 0.7 d [0.0–10.0 d] versus MR 1–2: 1.8 d [0.1–38.6 d] versus MR 0: 2.8 d [0.4–62.2 d], *p* < 0.01). The percent prolongation of pregnancy among the three groups was also shorter in women with severe inflammation (MR 3–4: 0.3%; MR 1–2: 0.8%; and MR 0: 1.4%, *p* = 0.038). Women with intact membranes had a longer pregnancy duration in the absence of inflammation relative to the women with “severe” or “minimal” inflammation (intact group, MR 3–4: 0.3 d [0–49.6 d] versus MR 1–2: 5 d [0.0–151.2 d] versus MR 0: 35.2 d [0.1–94.3 d], *p* < 0.001). Women with intact membranes who had “minimal” inflammation had significantly shorter pregnancies compared to those with “no” inflammation (*p* = 0.01). The percent prolongation among these three groups declined stepwise (MR 0: 15.1%; MR 1–2: 3.1%; and MR 3–4: 0.2%, *p* < 0.001).

### Relationships between Histological Evaluation of the Placenta for Acute Inflammation and the MR Score

We further determined that the presence and the severity of acute inflammation in the chorionic plate, amnion, chorio-decidua, and umbilical cord were significantly associated with the occurrence and the degree of intra-amniotic inflammation as determined by the MR score (Table 3). The MR score correlated significantly with the stages of chorioamnionitis (*r* = 0.599, *p* < 0.001). In multivariate regression analysis, both the MR score and the amniocentesis-to-delivery interval significantly predicted severity of chorio-amnionitis (*r* = 0.617, MR score: *p* < 0.001; amniocentesis-to-delivery interval: *p* = 0.04).

**Table 3:**
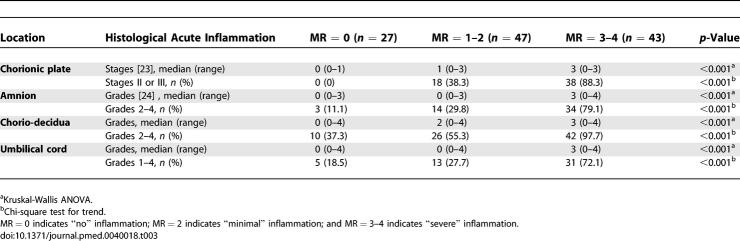
Prevalence and Distribution of Stages and Grades of Acute Inflammation in the Chorionic Plate, Amnion, Chorio-decidua, and Umbilical Cord Based on the “Severity” of Intra-amniotic Fluid Inflammation (*n* = 117)

### Relationships between MR Score and Early-Onset Neonatal Sepsis

Details on the neonate study population and hematological indices are given in Table 4. Four neonates had confirmed sepsis documented through a positive blood culture. Neonates delivered from mothers with AF MR 3–4 had significantly higher ABC (*p* < 0.001) and I:T ratio (*p* < 0.001), and increased incidence of suspected and/or confirmed sepsis (OR: 4.4 [95% confidence interval (CI), 1.7 to 11.6], *p* = 0.003) compared to neonates from mothers with MR 0 or MR 1–2 even after adjusting for GA at birth (ABC: *p* = 0.002; I:T ratio: *p* = 0.003). Suspected and confirmed sepsis in the neonatal period were associated significantly with an MR 3–4 (Chi-square = 8.270, Φ = 0.305, *p* = 0.004). The MR score maintained this association when adjusted for GA at birth (OR for MR 3–4: 3.3 [95% CI, 1.1 to 9.2], *p* = 0.03 and for GA at birth: 0.9 [95% CI, 0.8 to 1.0], *p* = 0.15). In multivariate logistical regression analysis with neonatal sepsis as a dependent variable and a panel of clinical (amniocentesis-to-delivery interval, clinical chorioamnionitis, GA at delivery, and PPROM) or AF biochemical characteristics as independent variables, the MR score, but not the WBC count, was the only variable with significant predictive value (MR 3–4: *p* = 0.03, WBC count: *p* = 0.48)

**Table 4:**
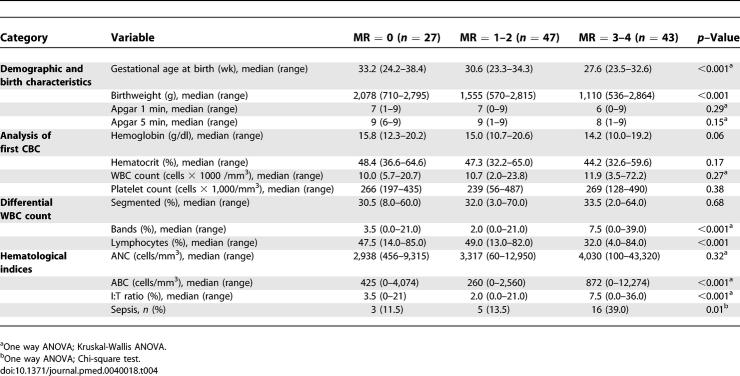
Characteristics and Hematological Indices of Neonates Admitted to the Newborn Special Care Unit (*n* = 104)

### Performance of the MR Score and Other Rapid Markers in Identification of Intra-amniotic Inflammation and Infection

Table 5 shows the estimates of the sensitivity, specificity, positive and negative predictive values, and accuracy of each method for the identification of women with intra-amniotic inflammation. The accuracy of an MR score of 3–4 was the highest (92.6%), followed by LDH and glucose (cutoff ≤ 10 mg/dl). An MR score of 3–4 had the strongest association with inflammation (Chi-square = 105.3, Φ = 0.822, *p* < 0.001), followed by LDH (Chi-square = 71.5, Φ = 0.754, *p* < 0.001). The MR score was originally designed to predict intra-amniotic inflammation (WBC > 100 cells/mm^3^) [14], but not to determine the number of WBCs that must be present for the MR score to change from a negative to a positive score. Using ROC analysis, we now find that a WBC cutoff of greater than 38 cells/mm^3^ predicts an MR score of 3–4. Thus, the MR score is actually more precise for the identification of intra-amniotic inflammation compared to the recommended WBC cutoffs. [16]. We next tested the utility of each test to predict a positive AF culture result (Table 6). The Gram stain had the highest specificity and accuracy for prediction of a positive AF culture. The MR score was less predictive of infection, consistent with its original design to assess inflammation. [14].

**Table 5:**
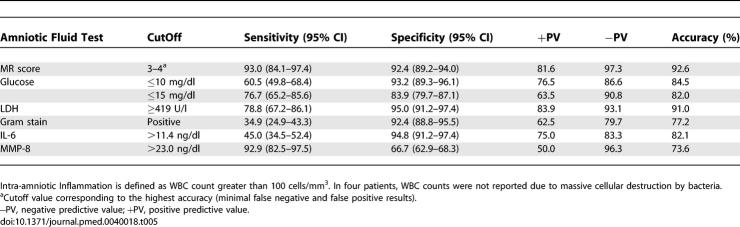
Performances of Laboratory Tests in Identifying Intra-amniotic Inflammation (*n* = 165)

**Table 6:**
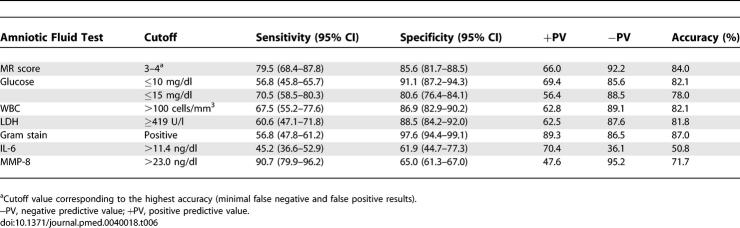
Performances of Laboratory Tests in Identifying a Positive Amniotic Fluid Culture (*n* = 169)

### Concordance among Tests to Diagnose Intra-amniotic Inflammation and Infection

Results from the five laboratory tests (glucose, Gram stain, LDH, IL-6, and MMP-8) were concordant in excluding inflammation in 64.7% of cases. In contrast, the MR score alone correctly excluded inflammation in 92.4% (*p* < 0.001) of cases. When inflammation was present, the five tests were concordantly positive in only 20.9%, whereas the MR score confirmed inflammation in 93.0% (*p* < 0.001).

We used stepwise logistic regression analysis to identify which clinical test or combination of tests optimally predicted inflammation, and found that the MR score performed better than any test or combination of tests (OR: 136.0 [95% CI, 42.0 to 632.3], *p* < 0.0001), followed by LDH (OR: 70.6 [95% CI, 20.7 to 240.7], *p* < 0.0001). We then asked whether addition of any other tests to the MR score could add significantly to its predictive value. When all covariates were entered into the model, only the MR score and LDH had a significant relationship with intra-amniotic inflammation (MR score 3–4: OR: 32.6 [95% CI, 6.5 to 164.1], *p* < 0.001; and LDH ≥ 419U/l: OR: 8.0 [95% CI, 1.7 to 38.6], *p* = 0.009). The addition of either IL-6 or MMP-8 to the model failed to improve the prediction of inflammation.

Stepwise logistic regression analysis for prediction of a positive AF culture revealed that the combination of Gram stain and MR score was identified as best in predicting a positive AF culture result (Gram stain: OR = 43.9 [95% CI, 9.6 to 200.5], and MR score: OR = 19.6 [95% CI, 6.5 to 59.0], *p* < 0.001). All the other covariates were excluded from the model based on the significance level *p* > 0.1. Addition of IL-6 and MMP-8 to the model did not improve the prediction value of an AF culture result.

When clinical variables such as maternal age, gravidity, parity, GA at amniocentesis, history of prior preterm delivery, cervical dilatation, and presence of uterine contractions or clinical chorioamnionitis were also introduced into the logistic regression model, none had significant or additive value for prediction of either infection or inflammation.

### Relationships between MR Score and Other Markers of AF Inflammation and Infection

Because AF inflammation is thought to be an important risk factor for neonatal morbidity and mortality, we tested the relationships between the AF MR score, the WBC count, IL-6 and MMP-8 levels, and glucose concentration in order to confirm our premise that MR scores of 1 and 2 reflect a lesser degree of inflammation, whereas an MR score of 3–4 indicates a more severe inflammation.

**Figure 2:**
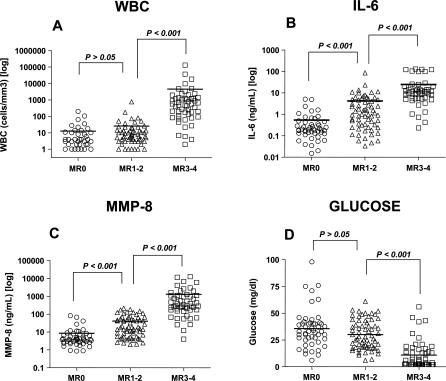
Proteomic Profiling of AF Versus Results of Other Diagnostic Tests of Intra-amniotic Inflammation and/or Infection (A) AF median WBC count, (B) ELISA for the IL-6 (C) and MMP-8 levels, and (D) glucose concentration in the same samples of AF (*n* = 169) varied with the degree of inflammation. Several degrees of inflammation were established using SELDI-TOF mass spectrometry (MR = 0 indicates “no” inflammation; MR = 2 indicates “minimal” inflammation; and MR = 3–4 indicates “severe” inflammation). Statistical comparisons were performed using Kruskal-Wallis ANOVA.

We found that women with MR 3–4 had higher (1) numbers of WBCs (median [range] (Figure 2A): MR 0: 3 [0–200] versus MR 1–2: 6 [0–767] versus MR 3–4: 532 [2–132,000] cells/mm^3^, *p* < 0.001); (2) levels of IL-6 (Figure 2B): (MR 0: 0.2 [0.0–5.1] versus MR 1–2: 1.1 [0.0–86.3] versus MR 3–4: 11.1 [0.2–129.6] ng/ml, *p* < 0.001); and (3) levels of MMP-8 (Figure 2C): (MR 0: 3.8 [0.9–82.0] versus MR 1–2: 17.7 [2.0–215.7] versus MR 3–4: 317.5 [4.0–12,644.58] ng/ml, *p* < 0.001). Amniotic fluid glucose concentration was lower in women with MR 3–4 (Figure 2D): (MR 0: 33 [6–98] versus MR 1–2: 28 [6–61] versus MR 3–4: 6 [2–56] mg/dl, *p* < 0.001). Because all the traditional tests for the detection of intra-amniotic inflammation/infection are characterized by a large variance, these findings indicate that only the MR score can be used to precisely identify the degree of inflammation present.

### Time Required Obtaining the AF Clinical Laboratory and Proteomic Results

The median time required to perform and report the final results for glucose and LDH levels was 48 min (range: 21–353) and 6.9 d (range: 2.1–24.4) for the AF cultures. The median time required to process, read, and score each AF sample per original protocol of the MR score [14] was 105 min (range: 65–161). However, in the current study, we determined that the minimum time of incubation required to obtain an accurate spectrum for defensin and calgranulin biomarkers was 15 min.

## Discussion

The results of this study indicate that proteomic profiling of the amniotic fluid is an accurate method for diagnosis of clinically relevant intra-amniotic inflammation. High MR scores are associated with preterm delivery, histological chorioamnionitis, and early-onset neonatal sepsis. Our research was motivated by the fact that validity from chance and bias may cause erroneous results and inflate expectations in observational proteomic research. To address such potential bias and to make the scientific community aware of the potential of new technology, it was critical that we design, conduct, and provide interpretation of our proteomic profiling in a totally unrelated population compared to the original study and in fresh samples of AF [14].

Clinical diagnosis of chorioamnionitis is an insensitive measure of the presence of inflammation, infection, or fetal sepsis [30,31]. Although to date, amniocentesis to investigate PTL or PPROM has not been demonstrated to have been of any clinical benefit in terms of the outcome, direct analysis of the AF remains the most accurate and direct method to assess for the presence or absence of intra-amniotic inflammation and infection. We found that proteomic profiling of the AF has the desired test characteristics for the detection of intra-amniotic inflammation, whether or not this occurs secondarily to infection [28]. Contrary to our initial investigation, we found that the interpretation of the MR score in the real world is not binary (“disease present” or “disease absent”), rather it is a gradient progressing from the absence of disease to “minimal” and then to “severe” inflammation. We demonstrated that the WBC count (one of the tests used for diagnosis of intra-amniotic inflammation) fails to effectively separate groups by their risk of preterm birth until the WBC level is very high. One does not need to be an obstetrician to understand how difficult it is in our current practice to interpret the results of the clinical tests routinely ordered to rule out infection and inflammation. Not only are the results often discordant, but their subjective interpretation based on previously reported diagnostic cutoffs for intra-amniotic inflammation frequently leads to iatrogenic preterm delivery in the absence of relevant confirmatory information [20].

Our concordance analysis clearly demonstrates the superiority and ease of interpretation of the MR score in comparison with any other clinical test judged alone or as a group. Proteomic profiling alone was able to correctly confirm or exclude inflammation in over 90% of our samples, whereas the other five tests taken together performed much less accurately. For clinical relevance, we compared the diagnostic performance of the MR score against glucose, WBC count, LDH, IL-6, and MMP-8 by using the clinical laboratory cutoffs previously reported in the literature [18,19,21,22]. Due to the retrospective nature of most of the previous reports, further studies need to be done to re-evaluate prospectively the cutoffs currently used by the clinicians to best diagnose intra-amniotic inflammation and infection, histological chorioamnionitis, and early-onset neonatal sepsis. Furthermore, such new cutoffs should be compared in a prospective fashion against the MR score.

We further demonstrated that, whereas Gram stain has the highest accuracy for the prediction of a positive AF culture, the MR score is additive and their combination predicts intra-amniotic infection long before the microbiological results are reported to the clinician. It should not be surprising that the MR score alone did not perform as well for the prediction of a positive culture, because it was designed to predict intra-amniotic inflammation [14], and there are causes of inflammation other than infection (e.g., decidual hemorrhage and immunologic diseases) [28].

Lastly, there was a significant association between a high MR score, placental acute histological inflammation, and both a shorter gestation and early-onset newborn sepsis. Our finding that the MR score, but not the absolute AF WBC count, is significantly related with early-onset neonatal sepsis, is provocative, and argues in support of the clinical utility of proteomic profiling.

From a pathophysiological perspective, the biomarkers comprising the MR score (defensins and calgranulins) are bioactive antimicrobial and anti-inflammatory proteins released following neutrophil activation [32,33]. We found that the components of a given MR score are not random, but recurrent and sequential. The current results suggest that the initial phase of the intra-amniotic inflammatory response is characterized by the appearance of defensins. Recognizing that the neutrophil count does not necessarily reflect functional responsiveness [34] and that neutrophil activation is a complex sequential event, it is reasonable to suggest that the generation of a proteomic fingerprint like the MR score provides a unique tool to monitor the degree of activation.

The median WBC count for the group of women with “minimal” inflammation (an MR score of 1 or 2) was extremely low (six cells/mm^3^). Currently, no clinical intervention can be recommended based on such a low WBC count. This argues for the clinical utility of performing proteomic analysis and not an AF WBC count. In addition, the results of our study seem to suggest that the MR score (one to two biomarkers present) can identify women at risk of premature delivery, when no clinical test other than proteomic profiling can do it. Thus, it is critical that at this stage, in women with “minimal” inflammation, we initiate as soon as possible prospective randomized trials to determine whether drugs with well-recognized anti-inflammatory properties (i.e., N-acetylcysteine) can halt the progress of diseases and prolong pregnancy [8]. Furthermore, several women with “severe” inflammation (an MR score 3–4) showed a full inflammatory response even when WBC counts were well below the currently recommended cutoffs for inflammation. This observation suggests that the concept of maternal and fetal inflammation needs revision, because the degree of neutrophil activation as reflected by the MR score may be more clinically relevant than the absolute WBC count.

Because early-onset sepsis is a risk factor for neonatal mortality and long-term neurodevelopmental outcome [1], recent advances in proteomics open opportunities for early recognition and treatment of the appropriate candidates [35]. In support of the proteomic profile, early-onset neonatal sepsis was associated significantly with an MR 3–4, but not with the AF WBC count. Certainly the findings of the current study are stimulating and set the stage for prospective randomized studies to determine the need, the duration of the antibiotic exposure of the newborn following delivery, and the long-term neurological outcome of the infant according to their AF MR score and or the treatment of choice made on the basis of the MR score.

One of the strengths of the present study in comparison with other studies is the inclusion of all amniocentesis samples in a prospective fashion rather than a predetermined selection of biological samples from diverse tissue banks. We also recognize the limitations of the investigation, as there is no antenatal clinical test that can be viewed as the gold standard for diagnosis of inflammation, and performance of a concurrent placental biopsy in utero to establish histological inflammation at the time of AF sampling is not possible. Despite this, our data indicate that the presence of biomarkers characteristic of intra-amniotic inflammation is related to the presence and the degree of acute histological inflammation at delivery. This again argues in support of the high accuracy of the MR scoring in detecting intra-amniotic inflammation as reflected by the AF WBC count, but also by the pathologic evaluation of the placenta.

In summary, in this study proteomic profiling of the AF was shown to be the most accurate test to identify women with intra-amniotic inflammation and assess the degree of neutrophil activation in the setting of preterm labor. Abnormal MR scores are associated with histological chorioamnionitis and early-onset neonatal sepsis.

## Supporting Information

### Accession Numbers

The SwissProt (http://www.ebi.ac.uk/swissprot) accession numbers for the proteins discussed in this paper are as follows: calgranulin A (P05109), calgranulin C (P80511), and neutrophil defensin-1 and neutrophil defensin-2 (P59665). Note: The accession numbers identify the unprocessed precursors of the biomarkers.

## Abbreviations

ABC: absolute band count
AF: amniotic fluid
ANOVA: analysis of variance
ELISA: enzyme-linked immunosorbent assay
GA: gestational age
CI: confidence interval
I:T ratio: immature/total neutrophil ratio
IL-6: interleukin-6
LDH: lactate dehydrogenase
MMP-8: matrix metalloprotease-8
MR score: Mass Restricted score
OR: odds ratio
PPROM: preterm premature rupture of membranes
PTL: premature labor
RBC: red blood cell
SELDI-TOF: surface-enhanced laser desorption ionization time-of-flight
WBC: white blood cell
